# Prediction of the Relative Resource Abundance of the Argentine Shortfin Squid *Illex argentinus* in the High Sea in the Southwest Atlantic Based on a Deep Learning Model

**DOI:** 10.3390/ani14213106

**Published:** 2024-10-28

**Authors:** Delong Xiang, Yuyan Sun, Hanji Zhu, Jianhua Wang, Sisi Huang, Haibin Han, Shengmao Zhang, Chen Shang, Heng Zhang

**Affiliations:** 1 Key Laboratory of Oceanic and Polar Fisheries, Ministry of Agriculture and Rural Affairs, East China Sea Fisheries Research Institute, Chinese Academy of Fishery Sciences, Shanghai 200090, China; xdl17852167218@163.com (D.X.);; 2 Laoshan Laboratory of Qingdao Marine Science and Technology Center, Qingdao 266104, China; 3 College of Marine Living Resource Sciences and Management, Shanghai Ocean University, Shanghai 201308, China; 4 College of Navigation and Ship Engineering, Dalian Ocean University, Dalian 116023, China

**Keywords:** *Illex argentinus*, southwest Atlantic, ensemble learning, deep learning, relative resource abundance

## Abstract

To analyze the impact of the marine environment on the relative abundance of *Illex argentinus* (high and low categories) in the southwest Atlantic, this study collected logbook data from Chinese pelagic trawlers from December 2014 to June 2024, including vessel position data and oceanographic variables such as sea surface temperature, 50 m and 100 m water temperature, sea surface salinity, sea surface height, chlorophyll-a concentration, and mixed layer depth. Vessel positions were used to enhance the logbook data quality, allowing an analysis of the annual trends in the resource center of this squid at a spatial resolution of 0.1° × 0.1° and a temporal resolution of ten days. The findings showed that the resource center is primarily located around 42° S in the north and between 45° S and 47° S in the south, with a trend of northward movement during the study period. Additionally, we constructed two ensemble learning models based on decision trees—AdaBoost and PSO-RF—aiming to identify the most critical environmental factors that affect its resource abundance; we found that the optimal model was the PSO-RF model with max_depth of 5 and n_estimators of 46. The importance analysis revealed that sea surface temperature, mixed layer depth, sea surface height, sea surface salinity, and 50 m water temperature are critical environmental factors affecting this species’ resources. Given that deep learning models generally have shorter running times and higher accuracy than other models, we developed a CNN-Attention model based on the five most important input factors. This model achieved an accuracy of 73.6% in forecasting this squid for 2024, predicting that the population would first appear near the Argentine exclusive economic zone around mid-December 2023 and gradually move east and south thereafter. The predictions of the model, validated through log data, maintained over 70% accuracy during most periods at a time scale of ten days. The successful construction of the resource abundance forecasting model and its accuracy improvements can help enterprises save fuel and time costs associated with blind searches for target species. Moreover, this research contributes to improving resource utilization efficiency and reducing fishing duration, thereby aiding in lowering carbon emissions from pelagic trawling activities, offering valuable insights for the sustainable development of this species’ resources.

## Simple Summary

*Illex argentinus* is an important economic species in the high seas of the southwest Atlantic, and its resource abundance and distribution are significantly influenced by changes in marine environmental factors such as water temperature and salinity. Therefore, this study employed an integrated learning model to analyze the importance of various marine environments on the resource abundance of this species. At the same time, this study also constructed a deep learning model and combined it with 2024 data to predict the abundance of the squid. After validation, the model was found to have high accuracy. In summary, this study conducted an accurate investigation into the impact of the marine environment on the squid and the prediction of its stock abundance. It can provide technical support for fishing operations of distant-water fishing companies, helping them reduce fishing costs, improve efficiency, and promote the sustainable harvesting of this squid resource.

## 1. Introduction

**Figure 1:**
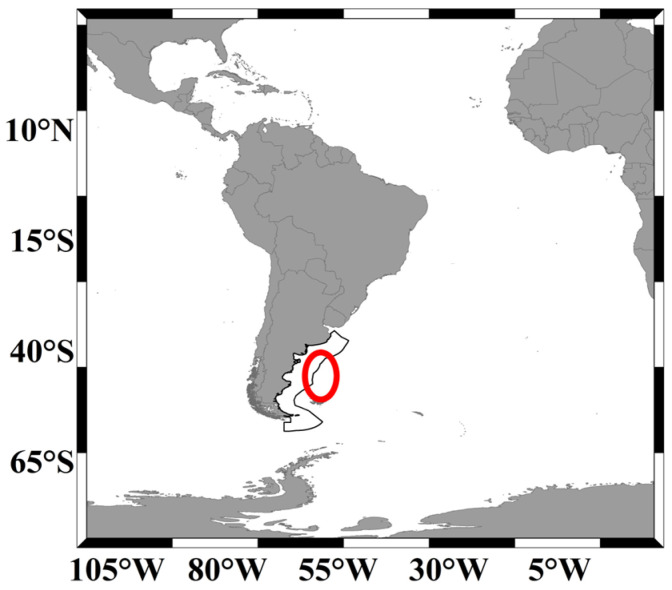
The main distribution area of *Illex argentinus* in the high seas of the southwestern Atlantic Ocean (the black solid line represents Argentina’s exclusive economic zone, and the red circles indicate the main distribution areas).

The southwest Atlantic is one of the regions in the world with high cephalopod fishery resource abundance [1]. The high sea’s fishing grounds are primarily located in the ocean area outside Argentina’s 200-nautical-mile exclusive economic zone (EEZ), on the Patagonian continental shelf (see Figure 1 below). This area is influenced by the convergence of the Brazil Current from the north and the Malvinas Current from the south [2]. It is also recognized as one of the most active regions for commercial fishing activities worldwide [3]. The convergence of warm and cold currents creates thermal fronts, which are often associated with high densities of marine biological resources. The Argentine shortfin squid, commonly known as *Illex argentinus*, is one of the main marine species in this region and is an economically important cephalopod [4,5]. This species belongs to the order Teuthoidea, family Ommastrephidae, and genus *Illex*. It is a pelagic, shallow-water species with a short lifecycle of approximately one year, typically not exceeding 18 months [6,7]. Due to its short lifespan and single-generation nature, this squid can respond rapidly to changes in the marine environment, and its habitat distribution is closely related to oceanic conditions [1]. This squid resource is primarily governed by Argentina’s national fishery authorities, which include the National Undersecretariat of Fisheries and Aquaculture, the Federal Fisheries Council, and the National Directorate for Fisheries Coordination and Supervision. To protect this resource, the authorities establish open and closed fishing seasons for squid jigging grounds, with a closed season for the high-sea squid jigging grounds from 1 September to 14 December each year [8]. However, there are currently no policies in place to set closed seasons for trawling grounds, allowing trawlers greater freedom in their operational periods. To ensure the sustainable long-term exploitation of this resource, the authorities follow Caddy’s approach of limiting fishing effort, adjusting it in the short term, and annually reducing the maximum proportion of harvestable biomass by 40% [9].

The reproductive behavior of this species exhibits distinct seasonal characteristics, with only one spawning event occurring throughout its lifecycle [10]. Females mature in about one month, while males take 1.5 to 2.5 months to mature [11], after which they die post-spawning. This biological trait results in populations that are primarily composed of a single generation, making resource fluctuations heavily dependent on recruitment, which can vary significantly due to various marine environmental factors such as water temperature, chlorophyll-a concentration, and salinity. Numerous studies have analyzed the impact of marine environments on squid fishing grounds and resource levels through different models, revealing that sea surface temperature (SST) is the most critical influencing factor [5,12,13]. SST has been validated as an important variable for fishing ground distribution in several models, acting in conjunction with other environmental factors like sea surface height (SSH) and sea surface salinity (SSS) to affect squid habitats and fishing areas. Additional research indicates that thermoclines also play a key role in the formation of fishing grounds, while the influences of SSH and SSS can be variable, sometimes not serving as key factors for identifying fishing grounds or habitats [14,15]. Moreover, combining CPUE and spatial overlay analyses determined that the optimal SST for squid is between 10 °C and 17 °C, with an optimal salinity range around 34‰, further validating the importance of SST in predicting fishing grounds [13].

The principle of forecasting resource abundance distribution refers to predicting the main distribution areas of a species and its temporal and spatial resource variations based on existing statistical biological distribution data and oceanographic environmental data. The magnitude of biological resource abundance is closely related not only to the area and distribution of habitats but also to population density [16]. High-quality forecasts of fish resource abundance in the ocean can help distant-water fishing enterprises improve their catch efficiency [17] and promote rational resource development. The primary environmental factors affecting squid resource abundance and fishing grounds include water temperature, salinity, and chlorophyll-a concentration. These environmental factors influence the behavior, feeding, and reproductive activities of species, thereby affecting the distribution of biological populations and habitat dynamics. Several studies have forecasted squid distribution using various models combined with marine environmental factors. Traditional forecasting models include Generalized Additive Models (GAMs) [18], Habitat Index Models [19], Bayesian Probability Models [20], and Markov Models [21]. With the rapid development of computer and artificial intelligence technologies, ensemble learning and deep learning techniques, such as Gradient Boosting Decision Trees [22], support vector machines [23], and Convolutional Neural Networks [24], are increasingly applied to predict habitat distribution, with overall accuracy rates around 70%. Fan [20] combined SST and other marine environmental data to build a Bayesian Probability Model to forecast the Pacific bonito fishing ground, and the comprehensive accuracy rate reached more than 70%. Cui [23] used the support vector machine model to predict the distribution of squid in the northwest Pacific Ocean, and the accuracy rate reached 57.4%. Han [24] built a Convolutional Neural Network model and combined with marine environmental data to predict the habitat distribution of Japanese mackerel in the northwest Pacific Ocean, and the accuracy rate reached 72%. Previous studies mostly used monthly time resolutions and spatial resolutions of 0.5° × 0.5°, which may overestimate the accuracy of forecasts. In actual fishing operations, fishermen require more refined and short-term forecasts of fishing grounds or resource abundance, yet such studies are rare. Additionally, since this squid is distributed both inside and outside the EEZ and squid-jigging vessels operate in both areas while trawlers can only operate outside the EEZ, most previous studies have used squid-jigging data. As a result, their findings may not provide accurate references for the operations of trawlers in the high seas, with the only notable work on the resource abundance and spatiotemporal variations of *Illex argentinus* in high-sea-trawling fisheries being by Xiang [5], who indicated that environmental factors such as SST, Chl-a, SSH, and 50 m water temperature significantly impact squid fishing ground distribution and have different contributions over time, but they did not predict or simulate high and low resource abundance. Compared to squid-jigging operations, trawling operations have higher yields. Meanwhile, the ocean currents in the high seas of the southwest Atlantic are strong and complex in direction. Trawling operations offer greater proactivity and flexibility and are less affected by sea conditions. Therefore, studying the distribution of squid resources in the trawling grounds of the high seas holds greater economic value.

The marine environment significantly influences the resource dynamics and distribution of *Illex argentinus*. Earlier predictions of this resource distribution relied on traditional models that could only handle limited environmental data. However, with advancements in computer science and improvements in hardware capabilities, current resource forecasting technologies have gradually become more diverse and complex. Concurrently, the development of remote sensing technology has increased the availability of oceanographic data. Machine learning and deep learning techniques, which can efficiently analyze the relationship between species distribution and environmental variables, also show considerable potential for growth. This study aims to (1) revise logbook data using vessel position information to enhance its scientific accuracy; (2) analyze the spatiotemporal variation characteristics of this squid’s resource abundance; (3) rank the importance of model factors using two ensemble learning models to identify key marine environmental factors affecting this species’ resource abundance; and (4) develop a forecasting model for resource abundance using deep learning techniques, exploring the trends in accuracy and influencing factors, thereby providing scientific evidence and practical guidance for understanding the changes and distribution of its resource abundance.

## 2. Materials and Methods

### 2.1. Data Sources

#### 2.1.1. Logbook Data and Vessel Position Data

In fishery research, the often-used metric of Catch Per Unit Effort (CPUE) serves as an indicator of the relative resource abundance of fish populations. A higher CPUE value indicates a greater resource abundance (i.e., higher fish density) in a given area, while a lower CPUE signifies lower resource abundance (i.e., lower fish density) [13]. The logbook data for this squid in the southwest Atlantic originate from Chinese trawlers operating in the high seas of this region from 2014 to 2024. The data include information such as the date of operation, positions for setting and retrieving nets, and catch quantities of various species. Since the primary operating area for this squid trawlers is located between 40° W and 50° W and 55° S and 60° S, this study focuses on fishing data from this region. The fishing season is defined as December of the previous year through June of the current year; for example, the 2021 fishing season spans from December 2020 to June 2021. The time resolution is set to biweekly, dividing each month into three ten-day segments: the first ten days (F), the second ten days (S), and the last ten days (L). For example, Jan/F refers to the first ten days of January. Each annual fishing season for this squid is categorized into three stages: early stage (early December to late December), peak stage (early January to late April), and late stage (early May to late June). By calculating the average catch per haul within 0.1° × 0.1° grid cells over the ten-year fishing season for each ten-day segment, we derive the CPUE values for those fishing grounds. The median CPUE is used as a threshold; areas above the median are classified as high-resource-abundance fishing areas, while those below the median are considered low-resource-abundance regions.

Vessel position data are sourced from the automatic identification system (AIS) and Vessel Monitoring System (VMS). The AIS is a radio-based automatic identification system that provides real-time, high-precision, and publicly accessible trajectory big data [25]. It serves as an open vessel data collection system and is a significant source of spatiotemporal data, collecting vast amounts of vessel position information [26]. Data frequency ranges from a few minutes to one hour; however, prior to 2019, limitations in satellite AIS network transmission capacity led to frequent data packet losses or the absence of AIS devices on some fishing vessels, resulting in missing or unreliable position data. The VMS refers to a ship monitoring system, which typically involves the installation of specific GPS reporting devices on vessels. These devices send position information via satellite networks to shore-based reception platforms, enabling real-time monitoring of vessels such as fishing boats. From 2014 to 2018, the data frequency was once every four hours, while from 2019 to 2024, it increased to once every hour. The vessel position data used in this study integrate both AIS and VMS data to ensure completeness and frequency that meet the quality correction needs of logbook data. These data include vessel name, timestamp, latitude and longitude, speed, and course heading. The fishing status is determined based on speed and heading, and the vessel position data are matched with the catch locations recorded in the logbook for each haul. This allows for the correction of inaccurate latitude and longitude information in the logbook based on the vessel position data. The vessel position data used in this study are all from trawlers, all of which operate with bottom trawling gear. The vessels range in length from 45 to 65 m, and there is no significant difference in fishing efficiency between them.

#### 2.1.2. Oceanographic Data

According to previous studies, factors such as sea temperature, salinity, chlorophyll-a concentration, and ocean currents can significantly influence the habitat of this squid [5,14]. Therefore, the oceanographic data utilized in this study include chlorophyll-a (Chl-a) data, sea surface temperature (SST) data, sea surface height (SSH) data, sea surface salinity (SSS) data, 50 m water temperature (50 m st), 100 m water temperature (100 m st), and mixed layer depth (MLD) data. The Chl-a data were downloaded from the website https://coastwatch.pfeg.noaa.gov (accesed on 1 September 2024), with a spatial resolution of 0.1° × 0.1°. Other oceanographic data were obtained from the Asia-Pacific Data Research Center (http://apdrc.soest.hawaii.edu/las_ofes/v6/dataset?catitem=71 (accesed on 1 September 2024)) and the Copernicus Marine Service (https://data.marine.copernicus.eu/products (accesed on 1 September 2024)), with the Asia-Pacific Data Research Center providing data at a spatial resolution of 0.1° × 0.1°, while the Copernicus Marine Service data have a spatial resolution of 0.083° × 0.083°. The study area selected for this research ranges from 35° W to 55° W and from 50° S to 70° S, with a temporal resolution set to ten days and a spatial resolution of 0.1° × 0.1°.

### 2.2. Data Processing Methods

#### 2.2.1. Revision of Catch Locations in Logbooks and Fishing Ground Centroids

The original logbook data contain some errors, likely due to recording mistakes made by the personnel during the production process. These errors mainly arise from discrepancies between the recorded vessel position at a specific time and the actual position of the vessel at that time. We revised the log data using vessel position information extracted from the AIS and VMS systems. First, we extracted the operation time for a specific haul from the logbook and then matched it with the corresponding data for the same vessel from the vessel position data, either at that time or at the nearest available time. We compared the latitude and longitude information from both the logbook and the vessel position data. Given that the operating speed of distant-water trawlers typically ranges from 3 to 6 knots and the duration of each operation lasts from 2 to 5 h, the range of the operating area is considerable, leading to some discrepancies. We set an error threshold of 0.3° (approximately 15 nautical miles), meaning that if the latitude and longitude difference between the log and vessel position data was within 0.3°, no revision was made. If it exceeded 0.3°, the latitude and longitude in the logbook data were replaced with those from the vessel position data. After revising the logbook data using vessel position information, 8.4% of the original log data had discrepancies exceeding the 0.3° threshold, indicating that 91.6% of the log entries were accurate and reliable. The accuracy of the revised log data exceeded 98%, making it suitable for model construction and validation.

After revising the log data, a weighted average calculation method was applied to compute the annual center of the squid fishing grounds. This approach was used to analyze the annual variation patterns of the squid, based on the principles outlined in Formula (1):

(1) $$X=\frac{\sum_{i=1}^{n}\left(C_{i}\times X_{i}\right)}{\sum_{i=1}^{n}C_{i}},Y=\frac{\sum_{i=1}^{n}\left(C_{i}\times X_{i}\right)}{\sum_{i=1}^{n}C_{i}}$$

In the formula, X and Y represent the latitude and longitude coordinates of the fishing effort centroid; X_i_ denotes the latitude of the ith net (in degrees); Y_i_ denotes the longitude of the ith net (in degrees); and C_i_ indicates the catch amount of the ith net (in tons) [14].

For the calculation of CPUE, we adopted a temporal resolution of 0.1° × 0.1°, calculating the average fishing effort for all fishing operations within each fishing area during each ten-day period, based on the principles outlined in Formula (2):

(2) $${CPUE}_{i,j,t}=\frac{\sum_{k=1}^{n_{i,j,t}}C_{i,j,t,k}}{\sum_{k=1}^{n_{i,j,t}}E_{i,j,t,k}}$$

In the formula, i, j represents the sequence of each fishing area defined by latitude and longitude, t denotes a specific time period, C_i,j,t,k_ is the catch amount (in tons) for the kth fishing operation during a specific ten-day period in that fishing area, and E_i,j,t,k_ corresponds to the fishing effort for the same operation. Additionally, n_i,j,t_ indicates the number of fishing operations in that area during the specified ten-day period.

After calculating the CPUE for each fishing area within each ten-day period, the median value was selected as the threshold. Utilizing the concept of relative resource abundance (RRA) [27], we categorized the areas into two groups: regions with CPUE greater than the median were defined as high RRA areas, while those with CPUE below the median were classified as low RRA areas.

#### 2.2.2. Ensemble Learning Models

After classifying the various grids, we extracted the corresponding marine environmental data and matched them with the grids. A single model may be susceptible to noise or imbalances in the dataset, which can lead to inaccurate assessments of feature importance. However, ensemble learning, by aggregating the decisions of multiple models, effectively reduces the bias and variance of individual models, providing a more robust evaluation of each feature’s contribution. Many ensemble learning methods can automatically calculate and output feature importance. They typically assess importance by measuring a feature’s contribution or influence on model split nodes, generating a feature importance ranking. Therefore, we employed two ensemble learning models, AdaBoost and Random Forest, to analyze the revised logbook data from 2015 to 2023. Using grid search and five-fold cross-validation, we selected the optimal model parameters. The grid search is a brute-force technique that tests all possible parameter combinations within a predefined range to find the best-performing combination on the validation set. Five-fold cross-validation is a common model evaluation method that divides the dataset into five non-overlapping subsets (folds). Each subset is used once as a validation set while the remaining four serve as the training set. This approach allows for more comprehensive data utilization, reducing performance fluctuations caused by different training and validation set divisions. By calculating the average performance, a more objective measure of the model’s effectiveness is obtained [28].

AdaBoost (Adaptive Boosting) is an ensemble learning algorithm introduced by Yoav Freund and Robert Schapire in 1996 [29]. As a boosting method [30], AdaBoost aims to enhance overall classification performance by combining multiple weak classifiers (weak learners) into a strong classifier [31]. Its main advantages include ease of implementation, a simple and efficient algorithm structure, resistance to overfitting, strong adaptability, and robust classification capability.

Random Forest, developed by Leo Breiman in 2001, is an ensemble learning algorithm based on decision trees. It constructs multiple decision trees [32] and combines their predictions for classification or regression tasks, thus improving the model’s generalization ability [33,34]. The advantages of Random Forest include strong resistance to overfitting, effectiveness in handling high-dimensional data, robustness to noise, and good scalability. To preprocess the population distribution data for this model, we employed the Particle Swarm Optimization (PSO) algorithm, introduced by Kennedy and Eberhart in 1995. In this approach, each particle represents a potential solution that moves through the solution space and updates its position based on feedback from the objective function [35,36,37]. Therefore, we incorporated the PSO algorithm into the Random Forest model to enhance its accuracy, resulting in the PSO-RF model.

The parameter settings for the two ensemble models primarily include max_depth and n_estimators (Table 1); in both models, n_estimators refers to the number of base models. Increasing n_estimators can improve generalization but may extend running time. max_depth controls model complexity; higher values extract more detail but risk overfitting, while lower values may limit the model’s expressiveness. By testing each parameter and comparing the accuracy, precision, recall, and F1-score of different models, we identified the optimal input parameters for the ensemble learning models. We then compared the performance of the two models using these optimal parameters and selected the best model to analyze the importance of various environmental factors. The importance of environmental factors refers to the relative weight of each factor’s impact on biological distribution, reproduction, and other behaviors. This importance can help identify which environmental factors play a crucial role in influencing biological populations [5], with a range of [0, 1] where higher values indicate greater importance. After analyzing the importance of each environmental factor, we selected the top five factors as the key environmental influences.

#### 2.2.3. CNN-Attention Model

**Figure 2:**
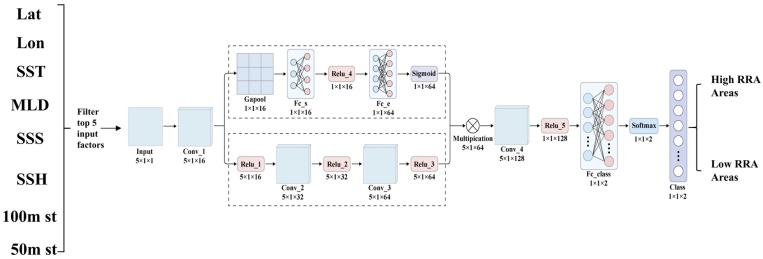
Structure diagram of the CNN-Attention model used in this study.

Due to the suitability of ensemble learning models for preliminary screening of suitable environmental features for fishing grounds and the ability of deep learning models to delve deeper into data characteristics, this study combines these two modeling approaches to enhance prediction accuracy. After identifying key marine environmental indicators affecting the resource abundance of *Illex argentinus* using ensemble learning models, we employed a Convolutional Neural Network (CNN) with an Attention mechanism to further construct a resource abundance forecasting model (see Figure 2 below). The CNN, a core architecture of deep learning, can extract raw features from training data and has strong nonlinear fitting capabilities. The basic structure includes convolutional layers, activation layers, pooling layers, and fully connected layers [38,39], aimed at automatically extracting features and classifying data. To address the potential redundancy of features from convolution operations [40], the proposed CNN-Attention model integrates five key model factors identified through ensemble learning, aiming to classify two types of marine resource abundance regions. The model input consists of spatiotemporal data for the five factors. After initial feature extraction through the convolutional layer, a feature map of 5 × 1 × 16 is generated. This feature map is then split into two parallel paths for further processing: one path reduces dimensions through global average pooling and passes through fully connected layers to output global environmental features; the other path captures deeper interactions between factors through convolutional layers. Ultimately, the outputs from both paths are multiplied element-wise to form a fused feature map, incorporating an attention mechanism to emphasize information relevant to resource abundance classification. After further high-level feature extraction through additional convolutional layers, the model outputs the classification results for two types of resource abundance areas. The evaluation of the deep learning model’s predictive performance will still utilize accuracy, precision, recall, and F1-score values. Additionally, we will use data from 2024 as the test set, with a temporal resolution of ten days. The predictive results for each ten-day period will be calculated, including the prediction accuracy for low-RRA areas (the number of accurately predicted low-RRA areas by the model divided by the total number of low-RRA areas predicted by the model), the prediction accuracy for high-RRA areas (the number of accurately predicted high-RRA areas by the model divided by the total number of high-RRA areas predicted by the model), and the overall accuracy (the number of accurately predicted RRA areas by the model divided by the total number of RRA areas predicted by the model).

## 3. Results

### 3.1. Fishing Ground Center of Gravity and CPUE

**Figure 3:**
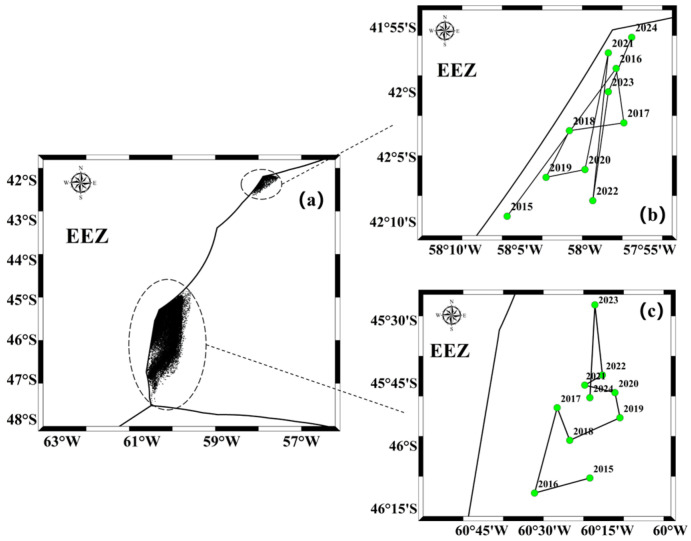
Changes in the fishing ground centroid of *Illex argentinus* habitat from 2015 to 2024 ((**a**) whole fishing location; (**b**) northern fishing ground; (**c**) southern fishing ground).

From 2015 to 2024, the resource distribution of this squid during the fishing season was statistically analyzed, and the annual centroid was calculated (see Figure 3 below). The resources of this species primarily distribute east of the exclusive economic zone line of Argentina in the southwest Atlantic Ocean, divided into northern and southern parts. The southern resources are mainly found near the exclusive economic zone line at 45° S to 47° S, while the northern habitat is primarily concentrated near the exclusive economic zone line at 42° S. According to the annual centroids calculated using Formula (1), the centroid of the southern resources has shown a trend of moving northward from 2015 to 2023, with a slight eastward shift, while in 2024, it moved southward. The centroid of the northern resources mainly distributes along the narrow shape of the exclusive economic zone line near 42° S.

**Figure 4:**
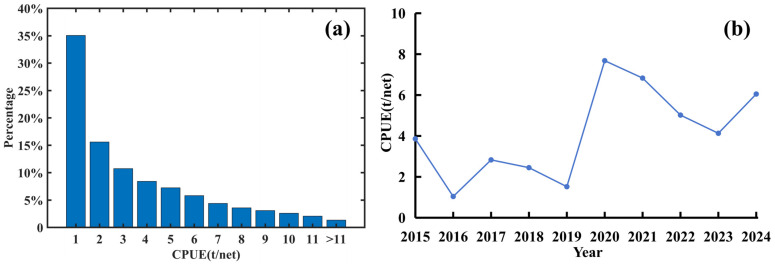
CPUE distribution and variation ((**a**) proportional distribution of CPUE values in *Illex argentinus* fishing areas from 2015 to 2024 (“1” indicates that the CPUE in the area ranges between 0 and 1 t/net; “2” indicates that the CPUE in the area ranges between 1.01 and 2 t/net); (**b**) annual variation trend of CPUE).

From 2015 to 2023, the average CPUE of this squid was highest at a ratio of 1 ton per net, and as the CPUE value increased, its proportion showed a gradually decreasing trend (see Figure 4 below). Based on the median CPUE, high and low resource abundance categories were established, finding that using 2 tons per net as the threshold for binary classification was appropriate. If the average catch of this squid in a fishing area grid during that period was 2 tons or more per net, the area was classified as a high-resource-abundance region (approximately 49.3%); conversely, areas with an average catch below 2 tons were classified as low-resource-abundance regions (approximately 50.7%). Based on the annual variation trend of CPUE, we found that CPUE was relatively low before 2020 and showed a significant increase starting from 2020. Additionally, CPUE exhibited noticeable fluctuations between years, reaching peaks in 2017 and 2020. Following these peaks, a downward trend was observed over the next two to three years, and after reaching the lowest CPUE in certain years, there was a marked increase in the following year.

### 3.2. Accuracy of Integrated Models and Identification of Key Environmental Factors

**Figure 5:**
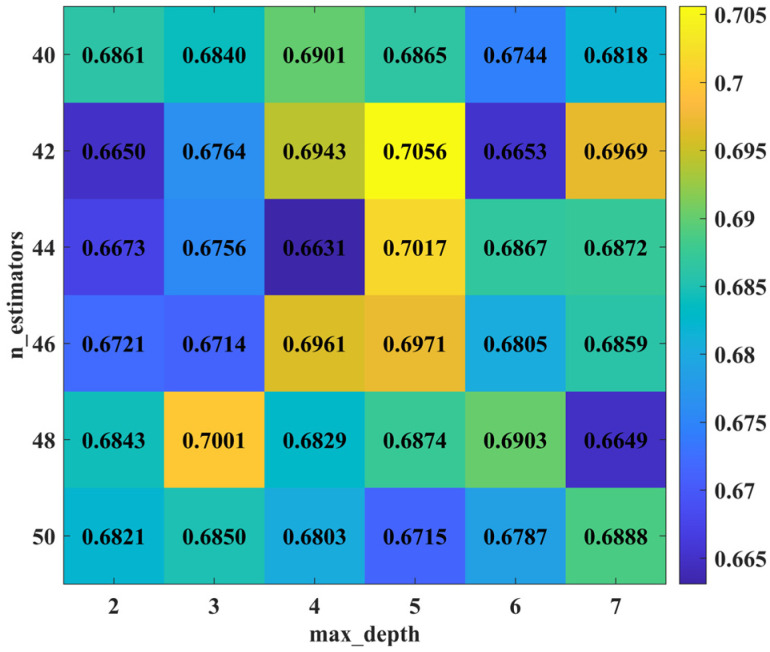
Changes in accuracy of AdaBoost model parameter combinations (max_depth ranging from 2 to 7 in increments of 1; n_estimators ranging from 40 to 50 in increments of 2).

After adjusting different parameters, the AdaBoost model achieved the highest accuracy of 0.7056 when max_depth was set to 5 and n_estimators was 42 (see Figure 5 below). Additionally, the model’s accuracy exceeded 0.70 when max_depth was 5 and n_estimators was 44, as well as when max_depth was 3 and n_estimators was 48. Therefore, it is determined that the optimal AdaBoost model is achieved at max_depth of 5 and n_estimators of 42.

**Figure 6:**
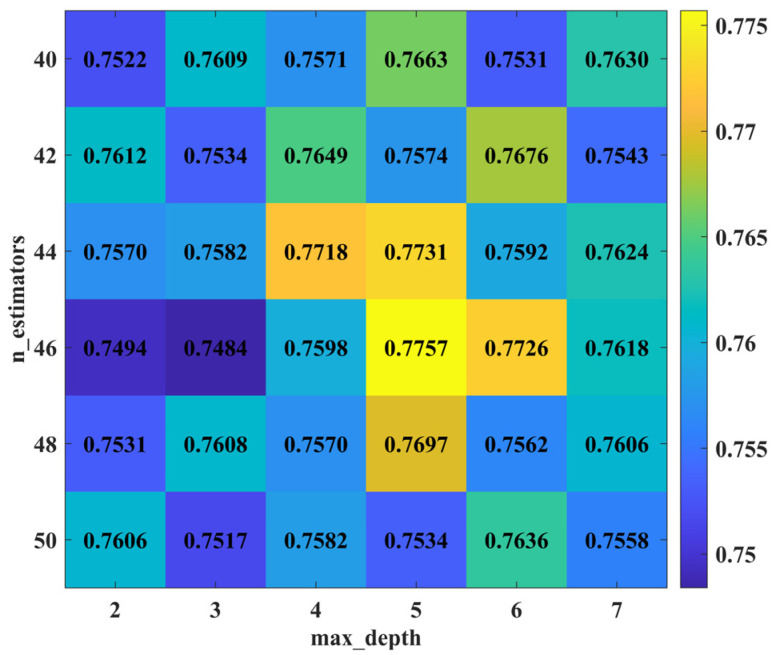
Changes in accuracy of PSO-RF model parameter combinations (max_depth ranging from 2 to 7 in increments of 1; n_estimators ranging from 40 to 50 in increments of 2).

After adjusting different parameters, the PSO-RF model achieved the highest accuracy of 0.7757 when max_depth was set to 5 and n_estimators qA 46 (see Figure 6 below). Additionally, accuracies exceeded 0.77 when max_depth was 4 with n_estimators at 44; max_depth was 5 with n_estimators at 44; and max_depth was 6 with n_estimators at 46. Therefore, it is determined that the optimal model for PSO-RF occurs at max_depth at 5 and n_estimators at 46. Comparatively, the accuracy of the optimal PSO-RF model is higher than that of the optimal AdaBoost model.

**Figure 7:**
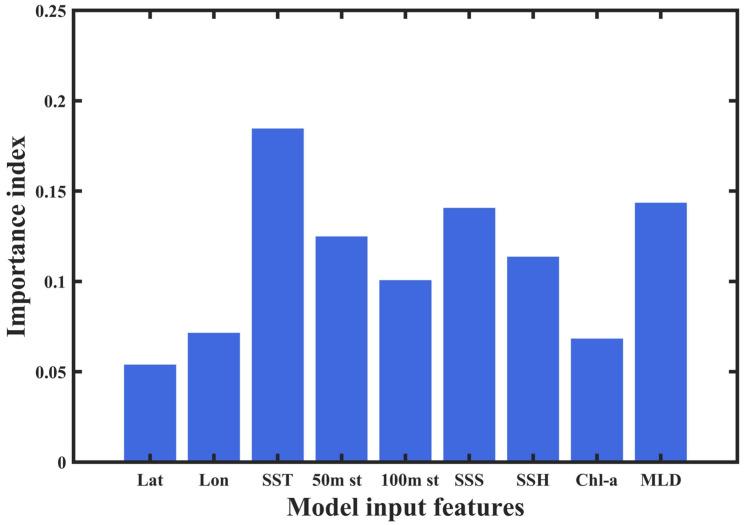
Importance of factors affecting the resource abundance of *Illex argentinus*.

At the same time, the precision, recall, and F1-score of the PSO-RF model (0.738, 0.742, and 0.740, respectively) were all higher than those of the AdaBoost model (0.667, 0.622, and 0.644, respectively). This indicates that the PSO-RF model provides superior analysis results for the resource abundance of *Illex argentinus* compared to the AdaBoost model. Therefore, the PSO-RF model was used to analyze the importance of each input factor (see Figure 7 below), with the importance ranking as follows: SST, MLD, SSS, 50 m st, SSH, 100 m st, Chl-a, Lon, and Lat. It can be observed that the marine environmental factor with the greatest impact on the habitat of this species is SST, followed by MLD. Based on the importance ranking, five key environmental factors with the highest importance were selected to construct the deep learning model.

### 3.3. CNN-Attention Model Accuracy and Validation

Compared to the PSO-RF model, the CNN-Attention model can automatically extract and learn both local and global features from the input data. In particular, when the data exhibit spatial or temporal correlations, the convolutional layers of the CNN can capture more complex feature interactions. The Attention mechanism enhances the model’s dynamic weighting ability, giving the CNN-Attention model a significant advantage in capturing complex patterns and multidimensional associations. Overall, while the RF model is suitable for preliminary feature selection, the CNN-Attention model delves deeper into data features, offering higher predictive accuracy and stronger model generalization. This modeling approach, combining RF and CNN-Attention, leverages both feature selection and complex feature learning, enhancing prediction accuracy.

**Figure 8:**
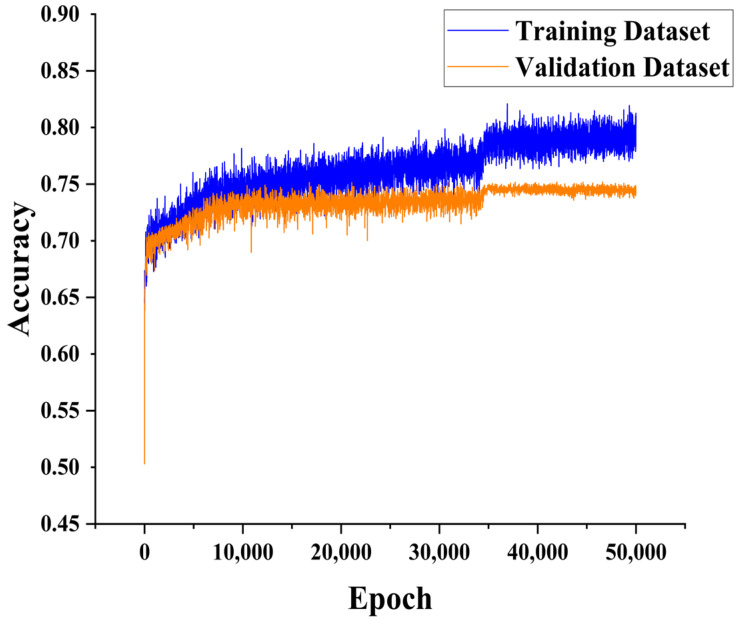
Accuracy of the training and validation sets of the CNN-Attention model.

**Figure 9:**
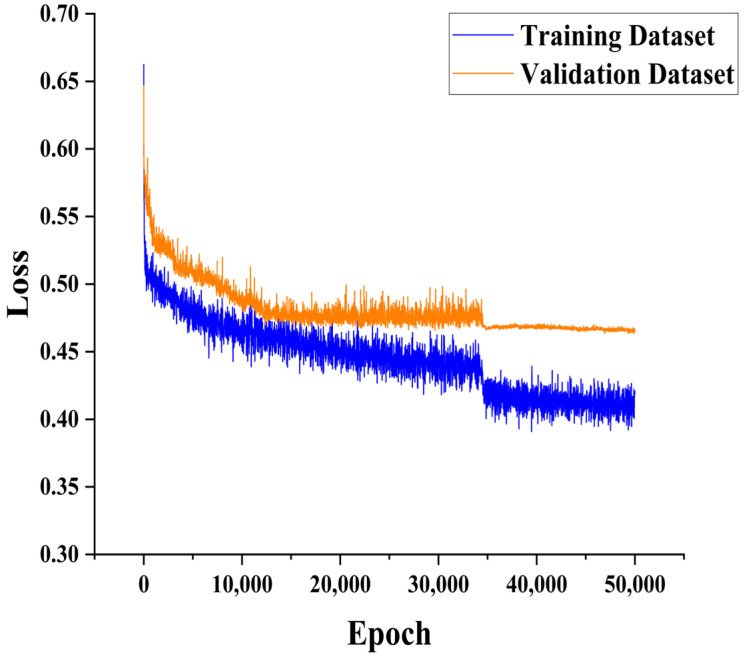
Loss of the training and validation sets of the CNN-Attention model.

In the early training stages of the CNN-Attention model, both training and validation accuracies increased significantly. After approximately 34,000 iterations, the accuracy and loss curves stabilized. Ultimately, after 50,000 training iterations, the model achieved an accuracy of 0.791 (see Figure 8 below). The trends in training accuracy and validation accuracy were consistent, indicating the good generalization ability of the model. The training loss and validation loss both showed significant decreases in the early stages of training, dropping below 0.44 and stabilizing after about 34,000 iterations. By the end of 50,000 training iterations, the loss decreased to 0.413 (see Figure 9 below). Additionally, the validation loss stabilized without an upward trend, indicating no significant overfitting.

**Figure 10:**
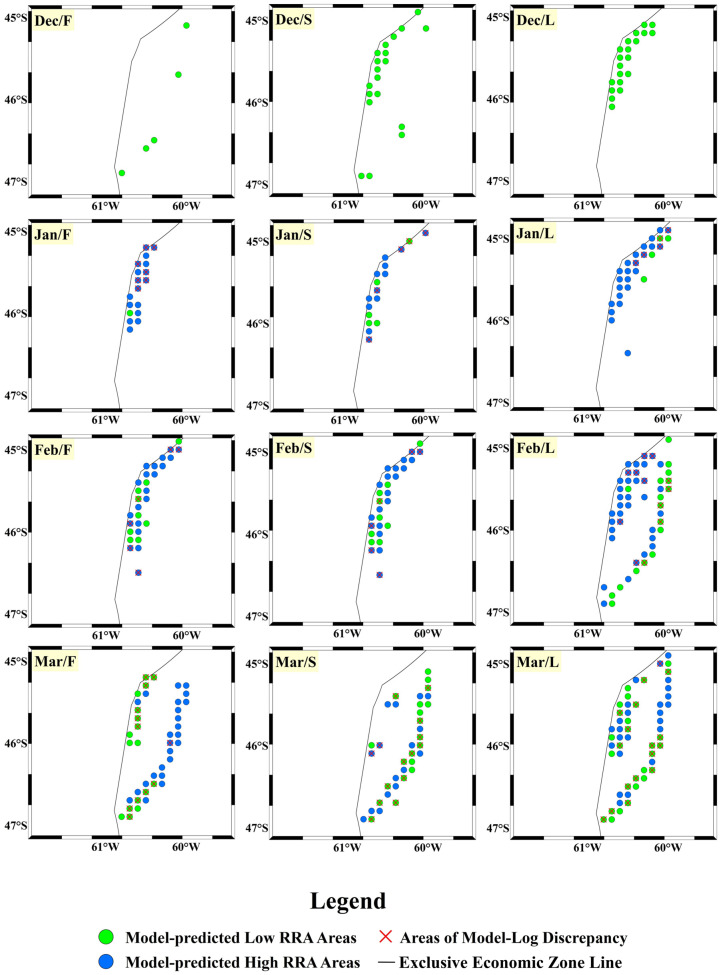
Habitat distribution maps predicted by the model and habitat distribution maps determined by logs from December 2023 to March 2024 (green circles indicate model-predicted low-RRA areas, blue circles indicate model-predicted high-RRA areas, and red crosses indicate areas where model predictions differ from log determinations).

**Figure 11:**
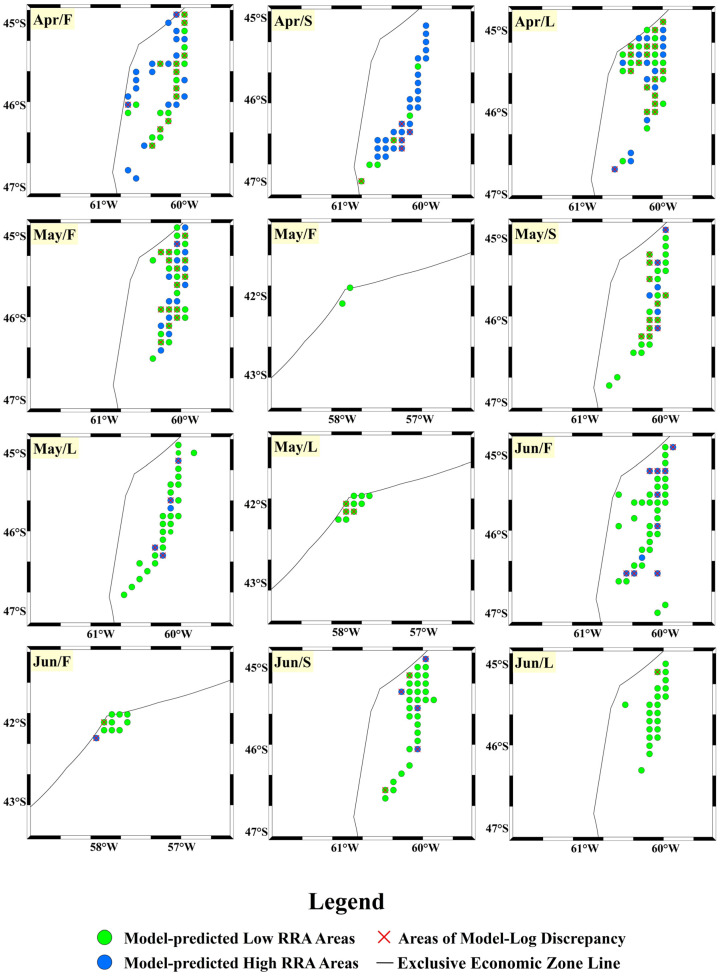
Habitat distribution maps predicted by the model and habitat distribution maps determined by logs from April 2024 to June 2024 (green circles indicate model-predicted low-RRA areas, blue circles indicate model-predicted high-RRA areas, and red crosses indicate areas where model predictions differ from log determinations).

**Figure 12:**
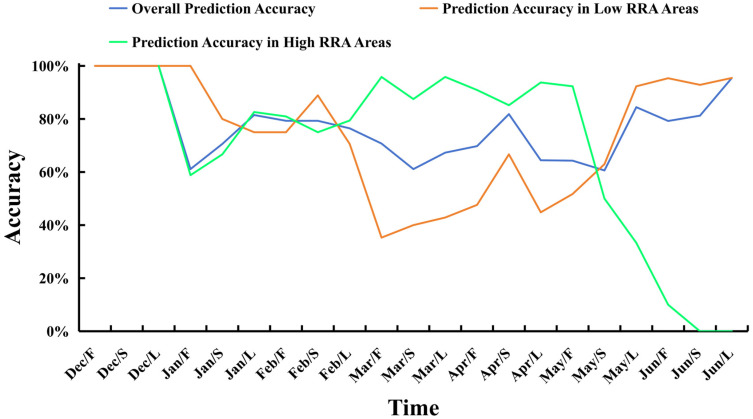
Accuracy of CNN-Attention model predictions for each period in 2024.

After completing the training of the CNN-Attention model, we imported the 2024 dataset into the model for predictions, using a time scale of ten days to forecast the relative resource abundance (RRA) of this species (see Figure 10 and Figure 11 below). We also calculated the accuracy of the predicted high-RRA and low-RRA areas, as well as the overall accuracy of the model (see Figure 12 below). In December 2023, no high-RRA areas were identified, indicating that the species population was likely concentrated within the exclusive economic zone (EEZ). Starting in January, high-RRA areas began to appear, with a substantial presence from January to April. In February, the high-RRA areas reached 1.46 times that of January. Although the high-RRA areas in March and April were slightly fewer than in February, they still reached 1.23 and 1.25 times that of January, respectively. After May/F, high-RRA areas began to decline sharply, with only 0.48 and 0.27 remaining in May and June. Additionally, we observed that the population of this species in the southwest Atlantic Ocean first appeared near the EEZ line at 45° S–46° S, with a slight northward movement from January to February. After Feb/L, the species began moving towards the southeastern deep-water areas, significantly expanding its habitat range. By May/L and Jun/F, the population moved close to the EEZ line at 42° S. The model’s accuracy reached 0.736, with a precision of 0.761, a recall of 0.665, and an F1-score of 0.710.

These metrics indicate that the model has good predictive performance. Analyzing the accuracy for each ten-day period, we found that in December and June, the model achieved high overall accuracy, with the highest prediction accuracy reaching 100% and the lowest at 79.2%. From January to May, the overall accuracy fluctuated between 60% and 90%, with the lowest accuracy occurring in May/S at 60.6% and the highest at 84.4% in May/L. Predictions for low-RRA areas exhibited a similar fluctuation trend, with lower accuracy between February and May, mostly below 70%, indicating that the model often misclassified high-RRA areas as low-RRA. In contrast, the predictions for high-relative-resource-abundance areas maintained a high accuracy for most of the time from January to April, often exceeding 85%. However, after May/S, the accuracy dropped sharply, likely due to the significant reduction in actual high-RRA areas, although the model continued to predict a small number of high-RRA regions (Figure 11).

## 4. Discussion

### 4.1. Center of Gravity of Illex argentinus Resource Abundance

Few scholars have conducted research on methods for correcting the quality of fishing log data. Properly evaluating and improving the quality of fishing log data can contribute to the objectivity and authenticity of fishery research. For example, Xie noted that the information recorded in nearshore light-trap fishery logs can be affected by operational conditions, weather, recording habits, and the level of understanding of fishing location and time standards of the skippers, leading to errors in the log data [41]. This study corrected the fishing log data using vessel position data and found that 8.4% of the log records contained errors exceeding 0.3°, which is obviously lower than the 20% error rate reported in other studies [42]. However, the accuracy of some records still needs to be considered. Nevertheless, 91.6% accuracy of the log data was found in the original fishing data, and the quality control log data (>98% accuracy) corrected by the vessel position data improved the precision of calculating the fishing ground center of gravity and matching with oceanic environmental data, enhancing the scientific value of this research.

In the early years of fishery development (1993–2010), the squid population was primarily distributed around 45° S to 47° S and near the Falkland Islands continental shelf [43]. Subsequently, the resource and fishing grounds of this species gradually expanded from the EEZ into international waters, with fishing practices evolving from squid jigging to a combination of jigging and trawling, leading to significant positional changes during each fishing season [43]. In the most recent years, from 2021 to 2023, the distribution of *Illex argentinus* trawl fishing grounds in international waters was found to exist in two main distribution areas, in the southern area (45° S to 48° S and 60° W to 62° W) and northern area (41° S to 43° S and 58° W to 60° W) [5]. In this study, using the revised fishing log data, the calculation of the squid habitat’s annual center of gravity also showed a clear trend of fluctuation, mainly along the eastern area of the Argentine exclusive economic zone (EEZ), which helps to more accurately identify the refined distribution of squid abundance and the central fishery operation area. According to CPUE statistics, the number of 0.1° × 0.1° small fishing areas with an average catch of less than 1 ton per net was highest, and with CPUE value increasing, the number of fishing areas showed an obviously decreasing trend. This indicates that high-resource-abundance areas are relatively dispersed throughout the study area. Using the median as a criterion for determining high-RRA-index areas aids in identifying relatively rich fishing resource zones, providing support for managers to formulate more refined fishing strategies [13]. Setting two tons per net as the threshold to distinguish between high and low RRA is reasonable, reflecting the low-density characteristics of squid resources in international waters and the dramatic fluctuations and periodic patterns over short time scales (weeks, months). Based on the calculation of the annual variation trend of CPUE, we found that the squid resource exhibits a clear cyclical pattern over the years. After reaching its peak, the resource quantity decreases year by year, but after two to three years of decline, the resource quantity quickly recovers. Currently, there is no indication of resource depletion for this squid, and its resource is relatively easy to recover, indicating its value for long-term fishing.

### 4.2. Importance of Environmental Factors

The Random Forest (RF) model demonstrates good performance in analyzing species distribution and the marine environmental factors affecting resource abundance, and it has been widely used by scholars in various countries. Qi [44] utilized the RF model and found that temperature, humidity, and wind are key environmental factors influencing the abundance of Spodoptera frugiperda in Africa. Dong [45] identified water depth and silicate as significant environmental factors affecting the distribution of soft coral Alcyonacea in the western Pacific using the RF model. In this study, by adjusting different parameters in the AdaBoost and PSO-RF models to find the optimal model, it was found that the performance of the PSO-RF optimal model surpassed that of the AdaBoost optimal model. This indicates that the PSO-RF model has better adaptability and performance in predicting the habitat of this squid. Further comparison of precision, recall, and F1-score revealed that the PSO-RF model significantly outperforms the AdaBoost model in these key evaluation metrics. Thus, we conclude that the PSO-RF model can more accurately capture the relationship between the squid’s habitat and its influencing factors, making it suitable for habitat analysis and prediction.

After selecting the optimal PSO-RF model, we analyzed the importance of each input factor. The results showed that sea surface temperature (SST) is the most significant factor affecting the habitat of this species, which is consistent with many existing studies [5,13,15]. This study found that the optimal SST range for the squid is 10.1 °C to 14.6 °C, with the most suitable SST typically occurring from January to April each year. However, after late May, when water temperatures drop below 9 °C, high-relative-resource-abundance (RRA) areas can still occur, possibly due to the squid’s increased adaptability to lower temperatures as they mature. Mixed layer depth (MLD), identified as the second most important factor, also indicates the impact of vertical water column structure on the habitat selection of the squid. The ocean mixed layer refers to the layer of mixed water in the ocean, where temperature and salinity distributions are usually more uniform, and energy and material exchanges between the ocean and atmosphere predominantly occur within this layer [46]. Gong [47] also found that MLD significantly affects the formation of squid fishing grounds in her study of the western Pacific. In the southwestern Atlantic region chosen for this study, which is relatively close to Antarctica and experiences cold and warm current interactions, the MLD varies significantly, ranging from less than 10 m to over 10 m. The optimal MLD ranges include several intervals: 12.9 m to 26.1 m, 30.8 m to 33.4 m, and 37.8 m to 40.4 m. The first interval primarily occurs from December to March each year, while the second and third intervals are mainly concentrated from April to June each year. In addition to the above two factors, other environmental factors also influence the habitat distribution of this squid. Sea surface salinity (SSS) affects the osmotic pressure balance within organisms, thereby influencing their metabolism [48]. This study found that the optimal salinity range for the squid is 33.8‰ to 34.1‰, indicating a narrow range, which suggests that the squid is sensitive to changes in salinity. Sea surface height (SSH) represents the convergence and divergence of ocean currents. Changes in SSH can promote the mixing of water bodies in the vertical direction, bringing rich nutrients and promoting primary productivity, thus affecting the formation of fishing grounds [18]. This study found that the optimal SSH range for the squid is −3.6 cm to −16.4 cm, which mainly occurs from December to March during the fishing season, with some distribution also seen from April to June. Additionally, this species exhibits vertical movement, inhabiting different depths of seawater, residing primarily in the bottom layer during the day and moving up near the surface at night [49]. The optimal ranges for the 50 m st and 100 m st factors selected in this study are 5.9 °C to 8.9 °C and 5.3 °C to 6.7 °C, respectively, with significant differences between the two during December to April and smaller differences in May and June. Chl-a concentration values indicate the strength of ocean primary productivity, with Chl-a being a fundamental indicator for estimating marine productivity, representing the abundance of phytoplankton in seawater [50]. This study found that the optimal range for Chl-a in high-RRA areas is mainly between 0.1 mg/m^3^ and 1.7 mg/m^3^, although high-RRA areas can still occur within higher Chl-a ranges. According to the PSO-RF results of this study, the importance of latitude and longitude is relatively low, indicating that this species is highly mobile due to its sensitivity to changes in the marine environment [51]. The importance of Chl-a is also low, suggesting that the squid does not directly feed on phytoplankton; Song Wei [52] found that it primarily consumes cephalopods and crustaceans, further supporting this conclusion. The importance of the 100 m st factor is also low, and due to seabed topography, there are many missing values for these data, with small differences between the 100 m st and 50 m st data. Ultimately, we selected the top five ranked environmental factors—SST, MLD, SSH, SSS, and 50 m st—to construct the CNN-Attention model. This operation helps the CNN-Attention model avoid incorporating weakly correlated variables, improving model efficiency, reducing training time, and lowering the risk of overfitting.

The CNN-Attention model’s predictions for 2024 also reflect the impact of key environmental factors on the resource abundance of this squid. In Dec/F 2023, the distribution range of the squid was minimal, with only low-RRA areas present, primarily due to low SST below 9.1 °C, which is significantly outside the optimal SST range. However, entering Dec/S, the five environmental factors fell within their optimal ranges, and the distribution range of the squid gradually increased. Nevertheless, high-RRA areas did not appear in late Dec/S and Dec/L, suggesting that large populations of the squid might still be residing within the waters of the Argentine exclusive economic zone. As January approached, high-RRA areas began to emerge and gradually expand, only to shrink after May, with a slight northward movement observed between January and mid-February. During Jan/F to Feb/S, SST primarily ranged between 11.2 °C and 14.2 °C, reaching the optimal SST range and sitting near its midpoint. This favorable SST was conducive to increased resource abundance for the squid. After entering Feb/L, SST dropped again to below 10 °C, and the squid population gradually moved southeast into deeper waters. This movement is closely related to their biological characteristics and seasonal migration patterns [53]. The water temperature at 50 m depth in the southeast was between 6.1 °C and 9.2 °C after February/L, indicating that the squid’s resource abundance remained high due to the optimal conditions at this depth. Studies have shown that after March, this species primarily inhabits deeper waters, whereas it was mainly found in shallower areas before this time [3]. Additionally, temperatures in deeper waters are lower than those in shallower areas, suggesting that individuals mature and adapt better to lower temperatures, often resulting in larger sizes in these habitats [54]. In this region, MLD was primarily between 12.1 m and 24.6 m before early Mar/F 2024, gradually increasing after Mar/S to exceed 60 m by Jun/L. SSS remained relatively stable, primarily concentrated between 33.8‰ and 34.0‰, falling within the optimal range. SSH was below −10 cm in Dec/F, but increased after Dec/L, remaining above −10 cm until Feb/S. After Feb/L, SSH decreased again, falling below −18 cm by Apr/L, exceeding the optimal SSH range. The variations in SSH exhibit a certain consistency with SST. According to our forecast for the resource abundance of this species in 2024, SSS remained within the optimal range. From December to February, the relative resource abundance of the squid was significantly influenced by changes in SST, MLD, and SSH. From March to April, the abundance was mainly affected by variations in 50 m st, MLD, and SSH. After May, as many environmental factors fell outside their optimal ranges, resource abundance gradually decreased [55].

### 4.3. Forecast Accuracy in the RRA Region 

To date, many studies have predicted squid fishing ground distribution and resource fluctuations in relation to marine environments. Wang [56] predicted the resource abundance of *Illex argentinus* in 2016 with a spatial resolution of 1° × 1° and a temporal resolution of one month, achieving an average accuracy of 90%. However, the coarse spatiotemporal scale may affect the precision of predictions in actual production and may be difficult to apply to actual fishing activities. Other studies have also forecasted squid fishing grounds; Cui [23] and Chen [57] used a spatial resolution of 0.5° × 0.5° and a monthly temporal resolution to predict the distribution of *Ommastrephes bartramii* in the northwest Pacific and purpleback flying squid (*Sthenoteuthis oualaniensis*) in the Indian Ocean, achieving average accuracies of 57.4% and 73.4%, respectively. The yearly average accuracy of the resource abundance forecast for this species reached 73.6% for 2024. The use of a finer spatial resolution of 0.1° × 0.1° and a temporal resolution of ten days makes this study more aligned with actual fishing needs for vessels compared to previous research. The distribution of high-RRA (relative resource abundance) and low-RRA regions was also analyzed. The results indicate that the distribution of this squid species exhibits significant spatiotemporal dynamics during the fishing season. According to the accuracy of the forecast results (Figure 12), predictions were relatively accurate during the early (Dec/F to Dec/L) and late (May/F to Jun/L) stage of the fishing season, while the accuracy during the peak stage (Jan/F to Apr/L) fluctuated. This suggests that in the early period, the resource quantity is low, making predictions easier, whereas during the peak season, resource abundance is higher, and the complexity of the marine environmental influences increases the difficulty of predictions. However, once mature, the squid’s adaptability to the environment improves, leading to higher model accuracy.

The accuracy of predictions for low-RRA regions exhibited a similar trend. In the early and late periods of the fishing season, the squid’s distribution mostly comprised low-RRA regions, with the model achieving high prediction accuracy, often exceeding 90% and even reaching 100% in many ten-day periods. During the peak season, the model occasionally predicted some high-RRA regions as being low-RRA, resulting in lower prediction accuracy for low-RRA areas, fluctuating between 35.3% and 88.9%. Conversely, the accuracy of predictions for high-RRA regions showed an opposite trend. In the early and late periods, few or no high-RRA regions were present, leading to lower accuracy (the accuracy for the early period was not calculated due to the absence of high-RRA regions), with many ten-day periods showing accuracy below 50%. After entering the peak period in Jan/F, various marine environmental factors in the area reached suitable levels for *Illex argentinus* [5], resulting in more prediction accuracy of high-RRA regions. From Feb/L, the extent of high-RRA regions peaked, reaching 1.7 times those at the beginning of the fishing season. During the peak fishing season (February to May), the accuracy of predicting high-RRA areas can reach over 90%. This indicates that the abundance of resources is relatively high during the peak fishing season [43].

## 5. Conclusions

This study revised the fishing log data of trawlers in the southwest Atlantic (2015–2024) using AIS vessel position data. By integrating these with oceanic environmental data, we trained two ensemble learning models (AdaBoost and PSO-RF) and identified the optimal one. Five key environmental factors influencing the relative resource abundance (RRA) of this squid were selected to analyze their impact, and a CNN-Attention deep learning model was constructed. The model effectively predicted the dynamic fluctuations in resource abundance for 2024, demonstrating pronounced spatiotemporal variability influenced by oceanic factors such as temperature. Evaluation metrics further indicate a high level of predictive accuracy and strong generalization capability. The environmental data utilized in this study can be acquired in near-real time and through relatively straightforward methods, which enhances the practicality and applicability of the research findings. These findings not only serve as a valuable reference for sustainable fishery management but also offer insights for addressing broader, critical challenges such as the impacts of climate change, the protection of marine ecosystems, and the optimization of fishing operations. By using these data, fishing plans can be adjusted in response to environmental changes, helping to reduce waste and overfishing. Additionally, this approach has significant potential in the fight against illegal, unreported, and unregulated (IUU) fishing, as it provides a clearer understanding of species distribution and behavior. Beyond *Illex argentinus*, the methodology and predictive models developed in this study can be adapted and applied to predict the habitats and resource levels of other marine species, thus offering a versatile tool for marine resource management and conservation efforts across various ecosystems.

Although this study has made some advancements in data revision and analytical methods, certain limitations remain. First, the revised data primarily addressed geographic coordinate corrections and did not fully account for potential errors arising from equipment malfunctions or human inaccuracies. Second, biological variables were not incorporated into the model. Given that this species exhibits varying adaptability to oceanic environmental factors across different maturity stages, future research should consider integrating biological data—such as size, weight, and gonad maturity—collected from different temporal and spatial contexts. Additionally, combining these with time-series data could improve the model’s ability to dynamically predict the squid’s habitat distribution.

## Figures and Tables

**Table 1:** Parameters in the Random Forest model.

Serial Number	Parameters	Range of Values
1	n_estimators	40, 42, 44, 46, 48, 50
2	max_depth	2, 3, 4, 5, 6, 7

## Data Availability

The marine environmental data used in this study can be downloaded from the following three websites: “https://coastwatch.pfeg.noaa.gov” (accessed on 1 September 2024), “http://apdrc.soest.hawaii.edu/las_ofes/v6/dataset?catitem=71” (accessed on 1 September 2024), and “https://data.marine.copernicus.eu/products” (accessed on 1 September 2024).
